# Tenecteplase versus alteplase for intravenous thrombolysis in acute ischemic stroke: a single-center propensity-weighted cohort study

**DOI:** 10.3389/fneur.2026.1928004

**Published:** 2026-09-25

**Authors:** Jin Ma, Heyireguli Wulayimu, Zhiqiang Ma

**Affiliations:** 1 The Seventh Affiliated Hospital of Xinjiang Medical University, Urumqi, China; 2 The Second Affiliated Hospital of Xinjiang Medical University, Urumqi, China

**Keywords:** acute ischemic stroke, alteplase, functional outcome, intravenous thrombolysis, inverse probability of treatment weighting, tenecteplase

## Abstract

**Background:**

Tenecteplase can be given as a single bolus, but comparative estimates from routine stroke care are susceptible to treatment-selection bias. Methods: We reviewed 269 adults treated at one center with intravenous tenecteplase (*n* = 110) or alteplase (*n* = 159) within 4.5 h of symptom onset from March 2024 through March 2026. The primary outcome was functional independence at 90 days [modified Rankin Scale (mRS) 0–2]. A logistic propensity-score model included pretreatment clinical and laboratory variables. Stabilized average-treatment-effect weights were truncated at the 1st and 99th percentiles. We report weighted risks, risk differences (RDs), odds ratios (ORs), and robust 95% confidence intervals (CIs). Secondary neurological/workflow and safety outcomes were adjusted within separate families using the Holm procedure.

**Results:**

After weighting, 90-day functional independence was 77.9% with tenecteplase and 81.3% with alteplase (RD, −3.5 percentage points; 95% CI, −17.0 to 10.1; *p* = 0.616; OR, 0.81; 95% CI, 0.36–1.82). Holm-adjusted *p* values for the other functional outcomes were 1.000. The weighted mean difference in door-to-needle time was −5.0 min (95% CI, −9.0 to −0.9; unadjusted *p* = 0.017; Holm-adjusted *p* = 0.068). Day-7 early neurological deterioration occurred in no tenecteplase recipient and nine alteplase recipients (weighted RD, −5.1 percentage points; 95% CI, −8.5 to −1.7; Holm-adjusted *p* = 0.016). SITS-MOST symptomatic intracranial hemorrhage occurred in four patients, with a weighted RD of 0.2 percentage points (95% CI, −1.7 to 2.1).

**Conclusion:**

Tenecteplase was not associated with greater 90-day functional independence in this propensity-weighted cohort. No clear between-group difference was observed in major hemorrhagic outcomes. DNT was shorter before multiplicity adjustment, and day-7 deterioration was less frequent after adjustment within the secondary neurological/workflow family.

## 1 Introduction

Intravenous thrombolysis remains standard reperfusion therapy for eligible patients with acute ischemic stroke who present within 4.5 h of symptom onset (1, 2). Alteplase requires a bolus followed by a 60-min infusion. Tenecteplase has greater fibrin specificity and a longer half-life and is administered as a single intravenous bolus.

AcT, TRACE-2, ORIGINAL, ATTEST-2, and TASTE compared tenecteplase 0.25 mg/kg with alteplase 0.9 mg/kg, and their findings support tenecteplase as an alternative thrombolytic in appropriately selected patients (3–8). Evidence from routine practice has also shown a low rate of symptomatic intracranial hemorrhage with the 0.25-mg/kg dose (9). Observational comparisons nevertheless require careful adjustment because drug availability, clinician preference, and stroke workflow can influence treatment choice.

We therefore estimated the association between thrombolytic agent and clinical outcomes in a single-center cohort, using inverse probability of treatment weighting (IPTW) to address measured pretreatment differences. Functional independence at 90 days was the primary outcome.

## 2 Materials and methods

### 2.1 Study design, setting, and reporting

This retrospective, single-center cohort study was conducted at the Second Affiliated Hospital of Xinjiang Medical University, Urumqi, China, and is reported in accordance with the STROBE recommendations (10). We identified consecutive adults who received intravenous thrombolysis within 4.5 h of acute ischemic stroke onset from March 2024 through March 2026. Data were obtained from the electronic medical record, stroke green-channel registry, laboratory information system, and imaging database. The final 90-day outcome assessment was completed on 3 June 2026. After follow-up and data checks were completed, the database was locked on 10 June 2026.

### 2.2 Participants

Among 421 patients screened during the study period, 152 did not meet eligibility for the predefined intravenous-thrombolysis-only analytic cohort, leaving 269 patients for analysis (Figure 1). Eligible patients were aged 18 years or older, had a clinical diagnosis of acute ischemic stroke, received tenecteplase or alteplase within 4.5 h, and had recorded pretreatment covariates, treatment-process variables, scheduled NIHSS assessments, and 90-day mRS. Exclusion criteria were a documented contraindication to intravenous thrombolysis, intracranial hemorrhage at presentation, severe bleeding diathesis, recent major surgery, intracranial tumor or vascular malformation, or absence of an essential propensity-score covariate or primary outcome. The final cohort comprised 269 patients: 110 received tenecteplase and 159 received alteplase. All included patients received intravenous thrombolysis alone; none underwent bridging therapy, mechanical thrombectomy, or another endovascular intervention.

**Figure 1:**
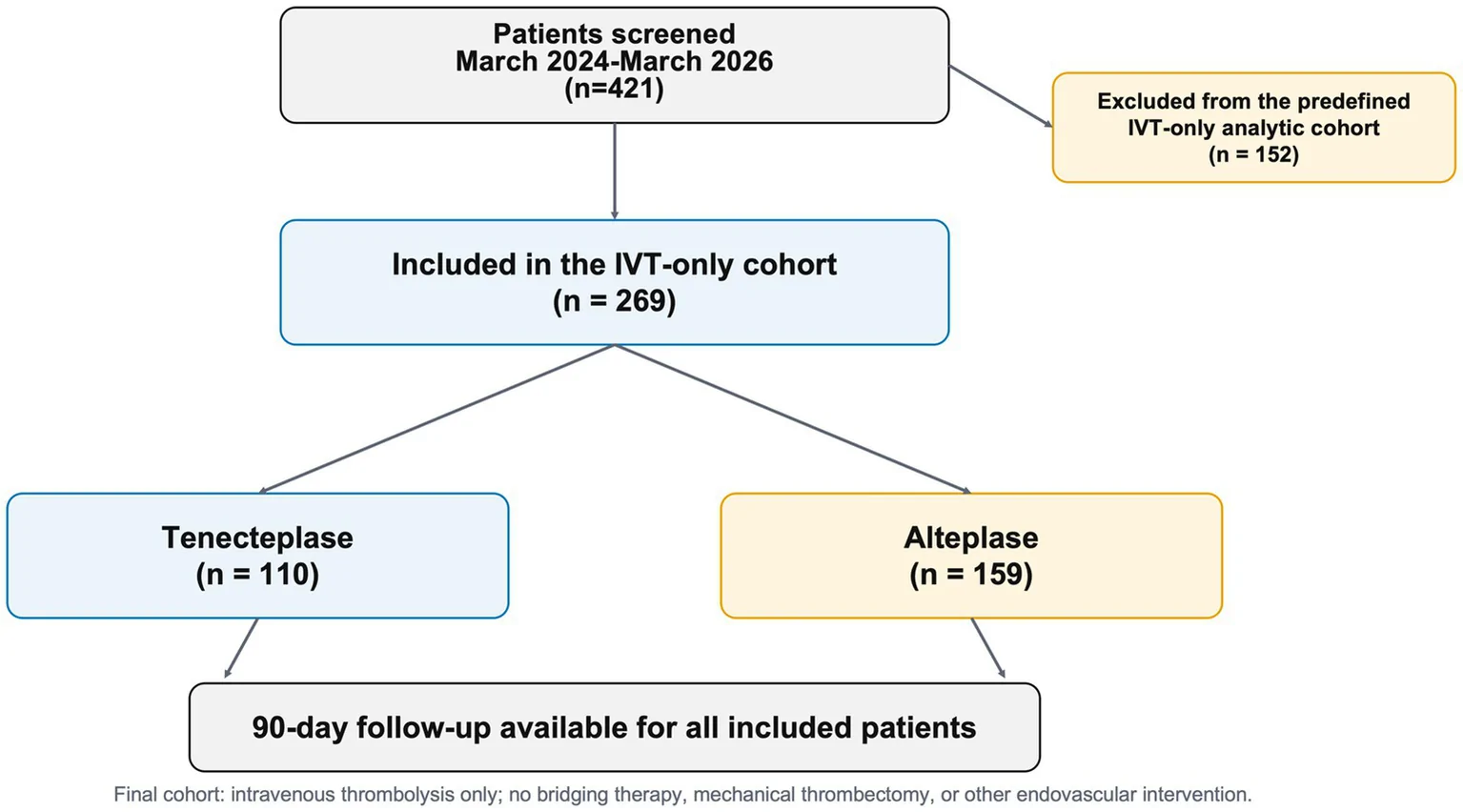
Participant flow. Among 421 patients screened, 152 were excluded from the predefined intravenous-thrombolysis-only analytic cohort and 269 were included. All included patients received intravenous thrombolysis alone without bridging therapy, thrombectomy, or another endovascular intervention.

### 2.3 Ethics approval and data governance

This retrospective observational study used routinely collected clinical records from patients treated at the Second Affiliated Hospital of Xinjiang Medical University. The study protocol was coordinated by the principal investigator affiliated with the Seventh Affiliated Hospital of Xinjiang Medical University and was reviewed and approved by the Medical Ethics Committee of the Seventh Affiliated Hospital of Xinjiang Medical University (approval no. 20251121–008; 21 November 2025). Clinical data were extracted by an investigator affiliated with the Second Affiliated Hospital and were de-identified before research analysis. The study involved no prospective intervention, collection of human biospecimens, or research-specific contact with participants. All study-specific data extraction and analysis were conducted after ethics approval. Written departmental authorization to use the de-identified clinical dataset for the approved research, manuscript revision, peer review, academic publication, and necessary verification was obtained from the Department of Emergency Medicine of the Second Affiliated Hospital of Xinjiang Medical University on 1 December 2025.

### 2.4 Thrombolysis protocol

Patients were evaluated through the institutional stroke green-channel pathway, including neurological examination, blood-pressure and blood-glucose assessment, laboratory testing when indicated, and noncontrast brain computed tomography. Tenecteplase was administered as a single intravenous bolus at 0.25 mg/kg (maximum 25 mg) over 5–10 s. Alteplase was administered at 0.9 mg/kg (maximum 90 mg), with 10% given as an intravenous bolus over 1 min and the remaining 90% infused over 60 min. These weight-based regimens were the institutional protocols used for the included treatments. Antiplatelet and anticoagulant therapies were withheld during the first 24 h unless clinically required; follow-up imaging preceded routine antithrombotic secondary prevention.

### 2.5 Variables, outcomes, and follow-up

Pretreatment variables were age, sex, body weight, admission systolic and diastolic blood pressure, hypertension, diabetes mellitus, atrial fibrillation, previous stroke, hyperlipidemia, coronary heart disease, current smoking, current alcohol use, cervical artery plaque, blood glucose, hemoglobin, platelet count, white and red blood cell counts, creatinine, international normalized ratio, prothrombin time, activated partial thromboplastin time, fibrinogen, alanine aminotransferase, TOAST subtype, baseline NIHSS, admission mRS, and onset-to-needle time. Stroke etiology was classified using the TOAST framework (11). Door-to-needle time (DNT) was treated as a post-arrival workflow outcome and was not included in the propensity-score model.

The prespecified primary outcome was functional independence at 90 days, defined as mRS 0–2; excellent outcome was mRS 0–1. Patients who died before follow-up were assigned mRS 6. Neurological status was measured with the NIHSS (12). Early neurological improvement was defined as a decrease of at least 4 NIHSS points from baseline or an NIHSS score of 0–1 at 24 h. The same definition was applied at day 7. Early neurological deterioration (END) was defined as an increase of at least 2 NIHSS points from baseline to the scheduled day-7 assessment. All remaining effectiveness, workflow, and safety outcomes were secondary.

Symptomatic intracranial hemorrhage (sICH) was defined according to SITS-MOST as local or remote parenchymal hemorrhage type 2 on imaging obtained 22–36 h after thrombolysis (or earlier when clinically indicated), accompanied by an increase of at least 4 NIHSS points from baseline or the lowest NIHSS within the first 24 h, or if the hemorrhage led to death (13). Asymptomatic intracranial hemorrhage (aICH) was any imaging-confirmed intracranial hemorrhage that did not meet the SITS-MOST sICH criteria. Alternative hemorrhage classification systems, including ECASS II and the Heidelberg Bleeding Classification, use different definitions and were not applied in the present analysis (21, 22). Other safety outcomes were mucocutaneous or gingival bleeding and all-cause death within 90 days.

The 90-day mRS was obtained at an outpatient visit or by structured telephone interview conducted by trained personnel (14, 15). Assessors were blinded to the thrombolytic agent. All 269 patients had recorded 90-day mRS and all required analysis variables; therefore, the analysis was complete-case without imputation.

### 2.6 Statistical analysis

Continuous variables are summarized as mean ± standard deviation or median (interquartile range), and categorical variables as n (%). A propensity score for tenecteplase was estimated using a main-effects logistic regression that included every pretreatment variable listed above. Stabilized IPTW targeting the average treatment effect was calculated as the marginal treatment probability divided by the individual propensity for the observed treatment. Weights were truncated at the pooled 1st and 99th percentiles. Positivity was evaluated from group-specific propensity-score ranges and an overlap histogram. Covariate balance was assessed using absolute standardized mean differences (SMDs), with SMD < 0.10 as the balance target; a full balance table and Love plot are provided (16). Effective sample size was calculated as (sum of weights)^2^/sum of squared weights and was not interpreted as a patient count.

Weighted marginal risks, RDs, and ORs were estimated for binary outcomes. Robust sandwich variances were used for weighted confidence intervals and tests. *p* values for the primary outcome and Holm adjustment within each outcome family were based on robust Wald tests of the RD. When a treatment group contained no event, inference emphasized the RD rather than an unstable OR (17). Weighted linear regression with robust variance estimated the mean DNT difference. The primary outcome was tested at a two-sided alpha of 0.05. Four secondary neurological outcomes (excellent outcome, 24-h early neurological improvement, day-7 neurological improvement, and day-7 END) and DNT comprised a five-outcome secondary neurological/workflow family. SITS-MOST sICH, aICH, mucocutaneous or gingival bleeding, and 90-day death comprised a four-outcome safety family. Each family was adjusted using the Holm procedure (18). Sensitivity analyses repeated the primary analysis using untruncated stabilized IPTW, 2.5th-97.5th percentile truncation, and overlap weights, which target patients with clinical equipoise (19). Analyses were reconstructed in Python 3.12.13 using pandas 2.2.3, NumPy 2.3.5, SciPy 1.17.0, and scikit-learn 1.8.0. Three verified extreme laboratory observations (fibrinogen 10.4 and 18.8, and red blood cell count 8.7) were retained in the primary analysis. A sensitivity analysis excluded the three corresponding records and re-estimated the propensity model and weights.

## 3 Results

### 3.1 Participants, overlap, and covariate balance

Of 421 patients screened, 152 were excluded from the predefined intravenous-thrombolysis-only analytic cohort and 269 were included (Figure 1): 110 received tenecteplase and 159 received alteplase. No analysis variable was missing. Estimated propensity scores ranged from 0.054 to 0.979 in the tenecteplase group and from 0.022 to 0.884 in the alteplase group (Figure 2). The raw stabilized weights ranged from 0.418 to 7.503; after truncation they ranged from 0.440 to 4.372. The group-specific effective sample sizes were 66.90 and 122.67, respectively. Weighting substantially reduced imbalance (Table 1; Supplementary Table S1; Figure 3). Diabetes mellitus (SMD = 0.110), red blood cell count (SMD = 0.107), and prothrombin time (SMD = 0.103) marginally exceeded the 0.10 threshold; all other reported pretreatment variables were below 0.10.

**Figure 2:**
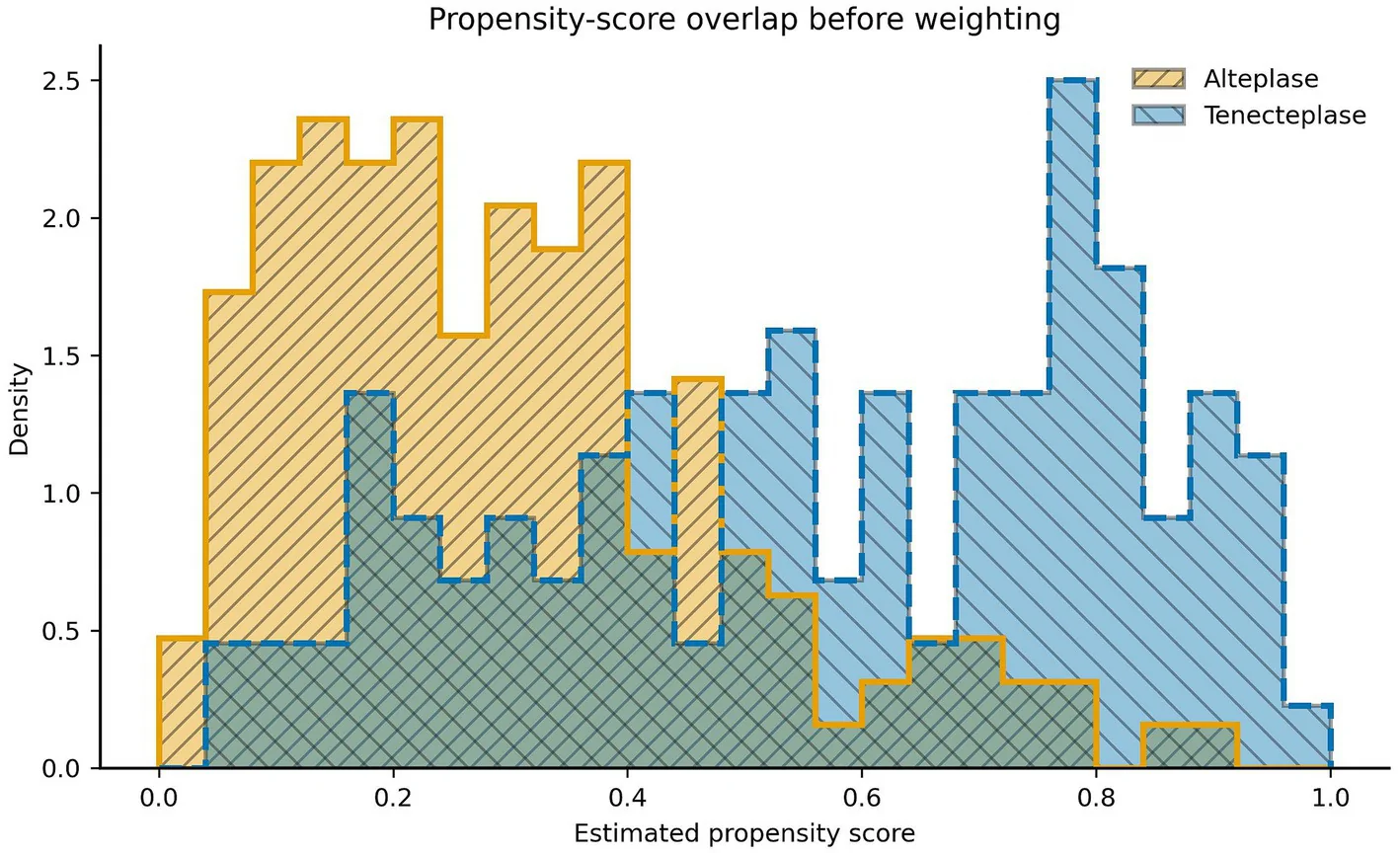
Propensity-score distributions by thrombolytic group before weighting. Color, opposing hatch patterns, and solid versus dashed outlines jointly distinguish the groups.

**Table 1:** Selected pretreatment characteristics and covariate balance.

Characteristic	Tenecteplase (*n* = 110)	Alteplase (*n* = 159)	Absolute SMD before IPTW	Absolute SMD after IPTW
Age	69.03 ± 11.23	65.01 ± 12.98	0.332	0.047
Systolic blood pressure	144.51 ± 22.53	145.96 ± 19.19	0.069	0.004
Diastolic blood pressure	82.11 ± 11.83	82.08 ± 14.47	0.002	0.015
Blood glucose	10.67 (7.98–14.77)	10.67 (9.09–13.07)	0.024	0.031
Fibrinogen	2.81 (2.44–3.32)	2.79 (2.41–3.28)	0.174	0.068
Baseline NIHSS score	4.00 (2.00–8.00)	4.00 (2.00–7.00)	0.019	0.006
Admission mRS score	2.50 (1.00–4.00)	3.00 (2.00–3.00)	0.036	0.061
Onset-to-needle time	106.00 (56.50–181.00)	137.00 (89.00–177.50)	0.082	0.027
Sex, *n* (%)	48 (43.6)	69 (43.4)	0.005	0.023
Hypertension, *n* (%)	88 (80.0)	119 (74.8)	0.124	0.046
Diabetes mellitus, *n* (%)	51 (46.4)	56 (35.2)	0.228	0.110
Atrial fibrillation, *n* (%)	9 (8.2)	20 (12.6)	0.145	0.025
Previous stroke, *n* (%)	3 (2.7)	4 (2.5)	0.013	0.036
TOAST: large-artery atherosclerosis, *n* (%)	42 (38.2)	57 (35.8)	0.048	0.033
TOAST: cardioembolism, *n* (%)	31 (28.2)	51 (32.1)	0.085	0.030
TOAST: small-vessel occlusion, *n* (%)	28 (25.5)	40 (25.2)	0.007	0.074
TOAST: undetermined etiology, *n* (%)	9 (8.2)	11 (6.9)	0.048	0.001

Values are mean ± SD, median (IQR), or *n* (%). The complete balance table for all propensity-score covariates is provided as Supplementary Table S1. DNT is excluded because it is a post-arrival workflow outcome. SMD, standardized mean difference; IPTW, inverse probability of treatment weighting; NIHSS, National Institutes of Health Stroke Scale; mRS, modified Rankin Scale; TOAST, Trial of ORG 10172 in Acute Stroke Treatment.

**Figure 3:**
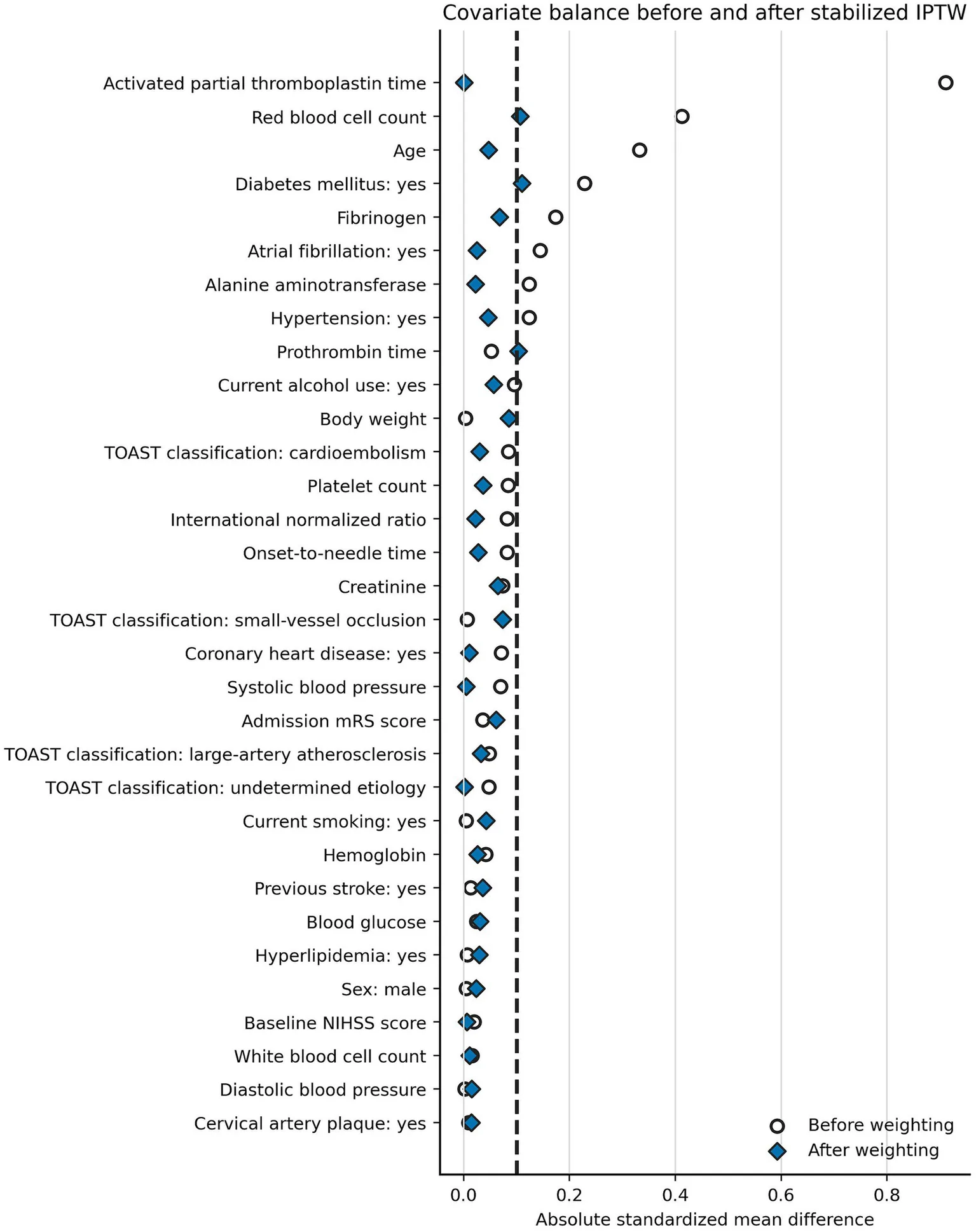
Covariate balance before and after stabilized IPTW. Open circles indicate values before weighting, blue filled diamonds indicate values after weighting, and the dashed line indicates an absolute SMD threshold of 0.10. Shape and fill preserve interpretability in grayscale.

### 3.2 Effectiveness, neurological, and workflow outcomes

The unweighted 90-day functional-independence rates were 83.6% (92/110) with tenecteplase and 82.4% (131/159) with alteplase. After IPTW, the corresponding marginal risks were 77.9 and 81.3% (RD, −3.5 percentage points; 95% CI, −17.0 to 10.1; *p* = 0.616; OR, 0.81; 95% CI, 0.36–1.82; Table 2; Figure 4). For excellent outcome, 24-h early neurological improvement, and day-7 neurological improvement, each Holm-adjusted *p* value was 1.000. No tenecteplase recipient and nine alteplase recipients met the day-7 END definition; weighted risks were 0.0 and 5.1% (RD, −5.1 percentage points; 95% CI, −8.5 to −1.7; secondary-family Holm-adjusted *p* = 0.016).

**Table 2:** Effectiveness, neurological, and workflow outcomes.

Outcome	Unweighted TNK	Unweighted alteplase	IPTW TNK % or mean	IPTW alteplase % or mean	RD, percentage points or mean difference (95% CI)	OR (95% CI)	*p*	Holm-adjusted *p*
90-day functional independence (mRS 0–2)	92/110 (83.6)	131/159 (82.4)	77.9	81.3	−3.5 (−17.0 to 10.1)	0.81 (0.36–1.82)	0.616	-
90-day excellent outcome (mRS 0–1)	77/110 (70.0)	110/159 (69.2)	64.0	69.1	−5.1 (−19.6 to 9.4)	0.80 (0.42–1.52)	0.492	1.000
Early neurological improvement at 24 h	42/110 (38.2)	58/159 (36.5)	39.3	36.1	3.2 (−11.1 to 17.5)	1.15 (0.62–2.10)	0.661	1.000
Neurological improvement at 7 days	72/110 (65.5)	111/159 (69.8)	62.1	68.5	−6.3 (−20.9 to 8.2)	0.76 (0.40–1.43)	0.394	1.000
Early neurological deterioration by day 7	0/110 (0.0)	9/159 (5.7)	0.0	5.1	−5.1 (−8.5 to −1.7)	–	0.003	0.016
Door-to-needle time, min	35.44 ± 12.17	40.81 ± 15.25	35.8	40.8	−5.0 (−9.0 to −0.9)	–	0.017	0.068

Actual patient counts are shown in the unweighted columns. Weighted columns report marginal percentages or means; counts were not reconstructed from weighted totals. For binary outcomes, p values and Holm adjustment use robust RD Wald tests; the DNT *p* value uses robust weighted linear regression. The OR is not reported for END because no event occurred in the tenecteplase group. RD, risk difference; OR, odds ratio; CI, confidence interval. Holm adjustment applies to the five secondary neurological/workflow outcomes; the primary outcome was not multiplicity-adjusted.

**Figure 4:**
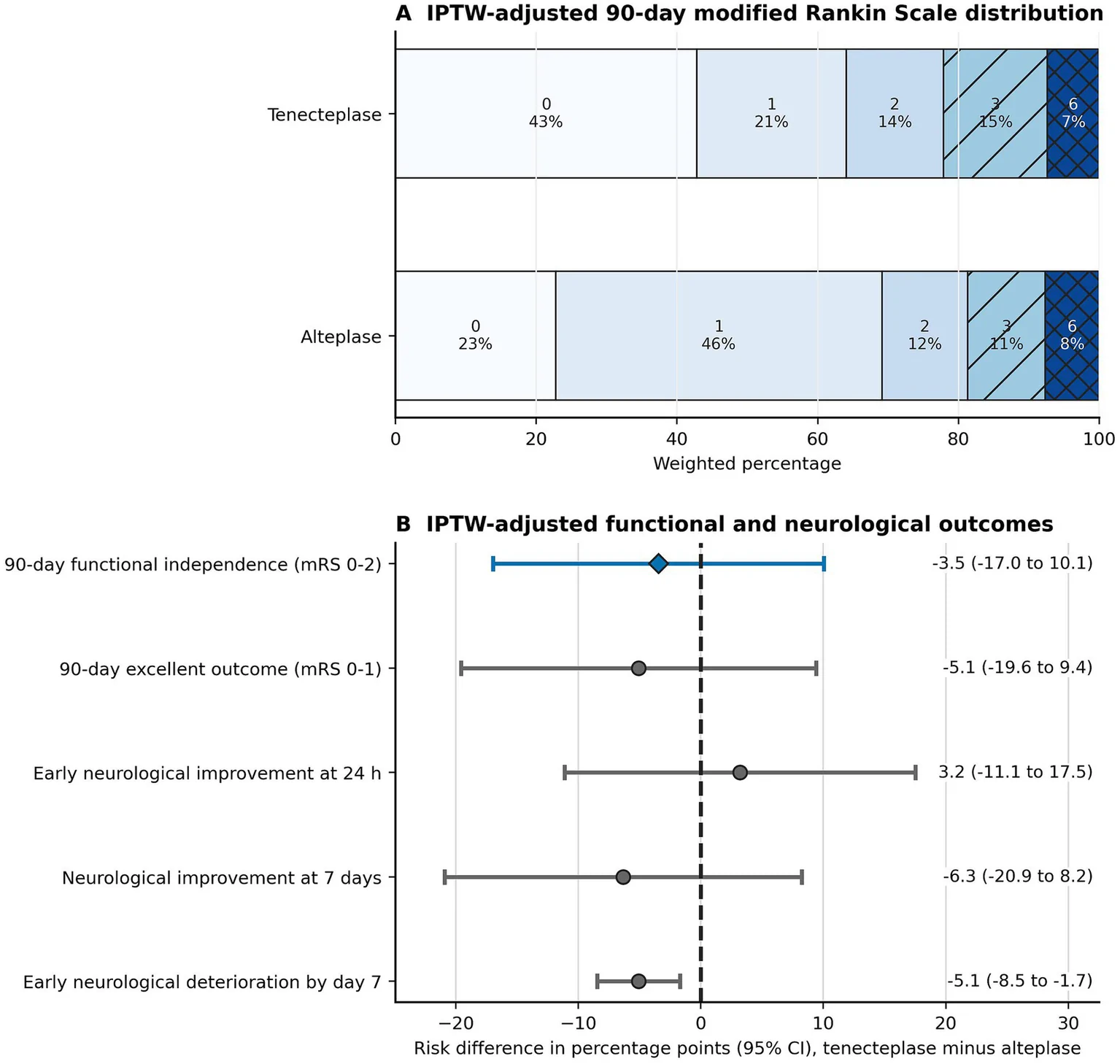
Functional and neurological outcomes. **(A)** IPTW-adjusted distribution of the 90-day modified Rankin Scale. **(B)** IPTW-adjusted absolute risk differences with robust sandwich 95% CIs; the diamond denotes the primary outcome.

Weighted mean DNT was 35.8 min with tenecteplase and 40.8 min with alteplase (mean difference, −5.0 min; 95% CI, −9.0 to −0.9; unadjusted *p* = 0.017; secondary-family Holm-adjusted *p* = 0.068).

### 3.3 Hemorrhagic and mortality outcomes

Four patients met the SITS-MOST sICH definition (2/110 tenecteplase and 2/159 alteplase); the weighted risks were 1.0 and 0.9%, respectively (RD, 0.2 percentage points; 95% CI, −1.7 to 2.1; safety-family Holm-adjusted *p* = 1.000). aICH occurred in 11/110 tenecteplase recipients and 33/159 alteplase recipients; the weighted risks were 9.3 and 18.4% (RD, −9.0 percentage points; 95% CI, −19.0 to 1.0; safety-family Holm-adjusted *p* = 0.305). The Holm-adjusted *p* values for mucocutaneous or gingival bleeding and 90-day mortality were both 1.000 (Table 3; Figure 5).

**Table 3:** Safety outcomes.

Outcome	Unweighted TNK	Unweighted alteplase	IPTW TNK %	IPTW alteplase %	RD, percentage points (95% CI)	OR (95% CI)	*p*	Holm-adjusted *p*
SITS-MOST symptomatic intracranial hemorrhage	2/110 (1.8)	2/159 (1.3)	1.0	0.9	0.2 (−1.7 to 2.1)	1.20 (0.16–8.73)	0.859	1.000
Asymptomatic intracranial hemorrhage	11/110 (10.0)	33/159 (20.8)	9.3	18.4	−9.0 (−19.0 to 1.0)	0.46 (0.17–1.27)	0.076	0.305
Mucocutaneous or gingival bleeding	4/110 (3.6)	3/159 (1.9)	2.5	1.6	0.9 (−2.3 to 4.1)	1.57 (0.33–7.45)	0.577	1.000
All-cause death within 90 days	7/110 (6.4)	13/159 (8.2)	7.4	7.7	−0.3 (−9.1 to 8.5)	0.95 (0.27–3.44)	0.943	1.000

Actual events are shown as n/N (%). Weighted percentages and robust RDs are emphasized because several outcomes were sparse. *p* values and Holm adjustment use robust RD Wald tests. sICH follows SITS-MOST; aICH is any imaging-confirmed intracranial hemorrhage not meeting sICH criteria. Holm adjustment applies across the four safety outcomes.

**Figure 5:**
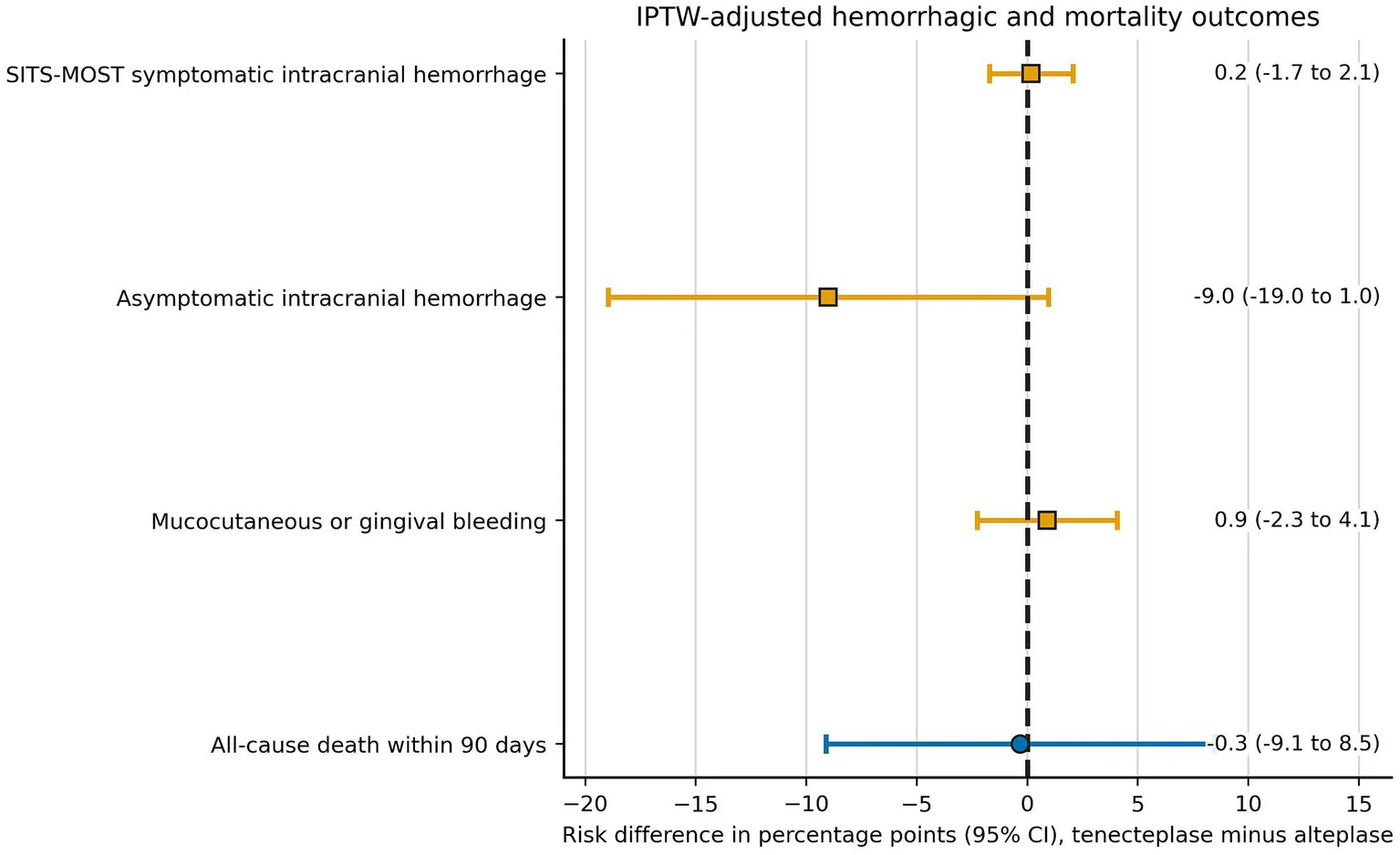
IPTW-adjusted absolute risk differences for hemorrhagic and mortality outcomes. Values are percentage-point differences with robust sandwich 95% CIs. Squares denote hemorrhagic outcomes and the circle denotes mortality.

### 3.4 Sensitivity analyses

The primary-outcome estimate was consistent across weight specifications (Table 4). The OR was 0.68 (95% CI, 0.28–1.65) with untruncated stabilized IPTW, 0.91 (95% CI, 0.42–1.97) with 2.5th-97.5th percentile truncation, and 0.99 (95% CI, 0.46–2.13) with overlap weighting. The overlap-weighted analysis achieved absolute SMDs below 0.10 for all modeled pretreatment covariates. After excluding the three verified extreme-laboratory records and re-estimating the propensity model and weights (n = 266), the weighted RD was −3.2 percentage points (95% CI, −16.8 to 10.4) and the OR was 0.82 (95% CI, 0.36–1.86).

**Table 4:** Sensitivity analyses for 90-day functional independence.

Weighting analysis	TNK risk, %	Alteplase risk, %	RD, percentage points (95% CI)	OR (95% CI)	*p*	ESS TNK	ESS alteplase
Stabilized ATE, untruncated	74.9	81.4	−6.5 (−22.2 to 9.3)	0.68 (0.28–1.65)	0.422	57.07	119.60
Stabilized ATE, 1st-99th percentile	77.9	81.3	−3.5 (−17.0 to 10.1)	0.81 (0.36–1.82)	0.616	66.90	122.67
Stabilized ATE, 2.5th-97.5th percentile	79.5	81.0	−1.5 (−13.8 to 10.8)	0.91 (0.42–1.97)	0.814	74.63	131.03
Overlap weights	81.2	81.3	−0.1 (−11.7 to 11.5)	0.99 (0.46–2.13)	0.989	82.06	113.92
Exclude 3 verified extreme-laboratory records; re-estimated PS and weights	77.9	81.1	−3.2 (−16.8 to 10.4)	0.82 (0.36–1.86)	0.643	66.62	120.15

The primary analysis used stabilized average-treatment-effect weights truncated at the 1st and 99th percentiles. Overlap weighting targets the population with clinical equipoise and achieved absolute SMD <0.10 for all modeled covariates. The extreme-value analysis excluded three verified records and re-estimated the propensity model and weights in 266 patients. ESS, effective sample size.

## 4 Discussion

The principal finding was that tenecteplase was not associated with a higher probability of 90-day functional independence after adjustment for measured pretreatment differences. Estimates for excellent outcome and scheduled NIHSS improvement at 24 h and day 7 were also close to the null. This is consistent with randomized evidence that supports tenecteplase as an alternative to alteplase but does not establish its superiority for functional outcome (3–8).

The weighted mean DNT was 5.0 min shorter with tenecteplase, a finding compatible with its single-bolus administration; the Holm-adjusted *p* value was 0.068. Day-7 END was less frequent with tenecteplase and remained significant after adjustment within the secondary neurological/workflow family.

Only four patients met the SITS-MOST definition of sICH, and the weighted RD was close to zero. Low absolute sICH rates with tenecteplase 0.25 mg/kg have also been reported in randomized trials and the CERTAIN collaboration (3–9).

The clinical contribution of this cohort is primarily practical. Current American Heart Association/American Stroke Association guidance supports either alteplase or tenecteplase for eligible patients treated within 4.5 h (20). In that context, the present estimates do not indicate a difference in 90-day functional independence, while the single-bolus regimen may simplify emergency workflow. The DNT association did not remain significant after multiplicity adjustment; the day-7 END result provides a clinically relevant signal for prospective evaluation.

### 4.1 Limitations

Several limitations affect interpretation. First, treatment was not randomized. IPTW reduced measured imbalance but cannot address unrecorded confounding, and diabetes, red blood cell count, and prothrombin time remained just above the SMD target. The number of modeled covariates and limited propensity-score overlap also reduced the effective sample size, particularly in the tenecteplase group. The analysis file did not contain individual treatment dates, preventing adjustment for treatment era, drug availability, or changes in stroke workflow and precluding a concurrent-period analysis. Large-vessel-occlusion and transfer-status data were unavailable. Although the included cohort was confirmed to have received intravenous thrombolysis alone without bridging therapy or thrombectomy, the findings cannot be generalized to patients selected for endovascular treatment or to a fully characterized large-vessel-occlusion population.

Second, baseline stroke severity was generally mild, which limits extrapolation to patients with severe stroke. The 90-day mRS was obtained through standardized outpatient or structured telephone assessments by assessors blinded to the thrombolytic agent; assessment mode was not available as a patient-level variable for a mode-specific analysis. END was reconstructed from the scheduled baseline and day-7 NIHSS values and could not capture transient deterioration between assessments. Finally, sparse events, particularly the four SITS-MOST sICH events, resulted in wide safety intervals. Secondary analyses also remain vulnerable to chance findings despite family-wise adjustment.

### 4.2 Strengths

The cohort was consecutive, and the primary outcome was available for every included patient. Outcomes were reconstructed from the recorded mRS and NIHSS fields using explicit rules. Weighted risks and RDs described sparse outcomes on an absolute scale, and the full balance diagnostics, effective sample sizes, and alternative weighting analyses support reproducibility of the propensity analysis.

## 5 Conclusion

In this single-center cohort, tenecteplase was not associated with greater 90-day functional independence after propensity weighting. No clear difference was observed in major hemorrhagic outcomes. Tenecteplase was associated with a shorter weighted DNT before multiplicity adjustment and less frequent day-7 END after adjustment within the secondary neurological/workflow family.

## Data Availability

The datasets presented in this article are not readily available because they contain sensitive clinical information and are subject to institutional and ethical restrictions. Requests to access the datasets should be directed to Zhiqiang Ma at kukuyixuesen@163.com and will be considered subject to approval by the relevant institutions and ethics committee.
