# Supplementary material for: Tenecteplase versus alteplase for intravenous thrombolysis in acute ischemic stroke: a single-center propensity-weighted cohort study
Source: Front Neurol. 2026 Sep 25;17:1928004. doi: 10.3389/fneur.2026.1928004 (PMC13649476; doi:10.3389/fneur.2026.1928004)
Supplement: Supplementary file 1 [file Supplementary_file_1.docx]

**Supplementary Material**

Tenecteplase versus alteplase for intravenous thrombolysis in acute ischemic stroke: a single-center propensity-weighted cohort study

# **Supplementary Table S1 Complete pretreatment covariate balance**

| **Characteristic** | **Tenecteplase (n=110)** | **Alteplase (n=159)** | **Absolute SMD before IPTW** | **Absolute SMD after IPTW** |
| --- | --- | --- | --- | --- |
| Age | 69.03 ± 11.23 | 65.01 ± 12.98 | 0.332 | 0.047 |
| Body weight | 67.53 ± 13.34 | 67.57 ± 11.85 | 0.004 | 0.085 |
| Systolic blood pressure | 144.51 ± 22.53 | 145.96 ± 19.19 | 0.069 | 0.004 |
| Diastolic blood pressure | 82.11 ± 11.83 | 82.08 ± 14.47 | 0.002 | 0.015 |
| Blood glucose | 10.67 (7.98-14.77) | 10.67 (9.09-13.07) | 0.024 | 0.031 |
| Hemoglobin | 138.23 ± 17.73 | 139.03 ± 20.34 | 0.042 | 0.026 |
| Platelet count | 212.30 ± 52.99 | 216.85 ± 55.90 | 0.084 | 0.036 |
| White blood cell count | 7.81 (5.75-10.12) | 7.29 (6.14-9.62) | 0.015 | 0.011 |
| Red blood cell count | 4.55 ± 0.46 | 4.78 ± 0.62 | 0.412 | 0.107 |
| Creatinine | 77.90 (52.95-106.38) | 72.20 (58.30-90.95) | 0.074 | 0.064 |
| International normalized ratio | 1.08 (0.99-1.15) | 1.06 (0.99-1.13) | 0.082 | 0.022 |
| Prothrombin time | 12.35 (11.10-13.60) | 12.00 (11.30-12.95) | 0.052 | 0.103 |
| Activated partial thromboplastin time | 26.00 (23.50-29.18) | 22.50 (20.00-24.40) | 0.911 | 0.001 |
| Fibrinogen | 2.81 (2.44-3.32) | 2.79 (2.41-3.28) | 0.174 | 0.068 |
| Alanine aminotransferase | 20.00 (13.25-35.75) | 23.60 (18.00-30.00) | 0.124 | 0.022 |
| Baseline NIHSS score | 4.00 (2.00-8.00) | 4.00 (2.00-7.00) | 0.019 | 0.006 |
| Admission mRS score | 2.50 (1.00-4.00) | 3.00 (2.00-3.00) | 0.036 | 0.061 |
| Onset-to-needle time | 106.00 (56.50-181.00) | 137.00 (89.00-177.50) | 0.082 | 0.027 |
| Sex, n (%) | 48 (43.6) | 69 (43.4) | 0.005 | 0.023 |
| Hypertension, n (%) | 88 (80.0) | 119 (74.8) | 0.124 | 0.046 |
| Diabetes mellitus, n (%) | 51 (46.4) | 56 (35.2) | 0.228 | 0.110 |
| Atrial fibrillation, n (%) | 9 (8.2) | 20 (12.6) | 0.145 | 0.025 |
| Previous stroke, n (%) | 3 (2.7) | 4 (2.5) | 0.013 | 0.036 |
| Hyperlipidemia, n (%) | 28 (25.5) | 40 (25.2) | 0.007 | 0.029 |
| Coronary heart disease, n (%) | 29 (26.4) | 47 (29.6) | 0.071 | 0.011 |
| Current smoking, n (%) | 39 (35.5) | 56 (35.2) | 0.005 | 0.042 |
| Current alcohol use, n (%) | 41 (37.3) | 52 (32.7) | 0.096 | 0.057 |
| Cervical artery plaque, n (%) | 42 (38.2) | 60 (37.7) | 0.009 | 0.015 |
| TOAST: large-artery atherosclerosis, n (%) | 42 (38.2) | 57 (35.8) | 0.048 | 0.033 |
| TOAST: cardioembolism, n (%) | 31 (28.2) | 51 (32.1) | 0.085 | 0.030 |
| TOAST: small-vessel occlusion, n (%) | 28 (25.5) | 40 (25.2) | 0.007 | 0.074 |
| TOAST: undetermined etiology, n (%) | 9 (8.2) | 11 (6.9) | 0.048 | 0.001 |

**Note.** Values are mean +/- SD, median (IQR), or n (%). All listed variables entered the main-effects propensity-score model. Admission mRS is the score recorded at admission; the original field label was corrected after source clarification. SMD, standardized mean difference; IPTW, inverse probability of treatment weighting.
